# A Circulating miRNA Signature for Stratification of Breast Lesions among Women with Abnormal Screening Mammograms

**DOI:** 10.3390/cancers11121872

**Published:** 2019-11-26

**Authors:** Sau Yeen Loke, Prabhakaran Munusamy, Geok Ling Koh, Claire Hian Tzer Chan, Preetha Madhukumar, Jee Liang Thung, Kiat Tee Benita Tan, Kong Wee Ong, Wei Sean Yong, Yirong Sim, Chung Lie Oey, Sue Zann Lim, Mun Yew Patrick Chan, Teng Swan Juliana Ho, Boon Kheng James Khoo, Su Lin Jill Wong, Choon Hua Thng, Bee Kiang Chong, Ern Yu Tan, Veronique Kiak-Mien Tan, Ann Siew Gek Lee

**Affiliations:** 1 Cellular and Molecular Research, Humphrey Oei Institute of Cancer Research, National Cancer Centre, Singapore 169610, Singapore; loke.sau.yeen@nccs.com.sg (S.Y.L.); gmsv1514@nus.edu.sg (P.M.); kohgeokling@gmail.com (G.L.K.); Claire.chan91@gmail.com (C.H.T.C.); 2 SingHealth Duke-NUS Oncology Academic Clinical Programme, Duke-NUS Medical School, Singapore 169857, Singapore; madhukumar.preetha@singhealth.com.sg (P.M.); benita.tan.k.t@singhealth.com.sg (K.T.B.T.); yong.wei.sean@singhealth.com.sg (W.S.Y.); sim.yirong@singhealth.com.sg (Y.S.); lim.sue.zann@singhealth.com.sg (S.Z.L.); juliana.ho.t.s@singhealth.com.sg (T.S.J.H.); james.khoo.b.k@singhealth.com.sg (B.K.J.K.); thng.choon.hua@singhealth.com.sg (C.H.T.); veronique.tan.k.m@singhealth.com.sg (V.K.-M.T.); 3 Division of Surgical Oncology, National Cancer Centre, Singapore 169610, Singapore; thung.jee.liang@nccs.com.sg (J.L.T.); ong.kong.wee@singhealth.com.sg (K.W.O.); oey.c.l@nccs.com.sg (C.L.O.); 4 Department of General Surgery, Singapore General Hospital, Singapore 169608, Singapore; 5 SingHealth Duke-NUS Breast Centre, Singapore 169610, Singapore; 6 Department of General Surgery, Sengkang General Hospital, Singapore 544886, Singapore; 7 Department of General Surgery, Tan Tock Seng Hospital, Singapore 308433, Singapore; patrick_chan@ttsh.com.sg (M.Y.P.C.); ern_yu_tan@ttsh.com.sg (E.Y.T.); 8 Division of Oncologic Imaging, National Cancer Centre, Singapore 169610, Singapore; jill.wong.s.l@singhealth.com.sg; 9 Department of Diagnostic Radiology, Tan Tock Seng Hospital, Singapore 308433, Singapore; chong_bee_kiang@ttsh.com.sg; 10 Department of Physiology, Yong Loo Lin School of Medicine, National University of Singapore, Singapore 117593, Singapore

**Keywords:** circulating microRNAs, breast cancer, molecular diagnosis, detection, stratification, mammography, blood-based test, liquid biopsies

## Abstract

Although mammography is the gold standard for breast cancer screening, the high rates of false-positive mammograms remain a concern. Thus, there is an unmet clinical need for a non-invasive and reliable test to differentiate between malignant and benign breast lesions in order to avoid subjecting patients with abnormal mammograms to unnecessary follow-up diagnostic procedures. Serum samples from 116 malignant breast lesions and 64 benign breast lesions were comprehensively profiled for 2,083 microRNAs (miRNAs) using next-generation sequencing. Of the 180 samples profiled, three outliers were removed based on the principal component analysis (PCA), and the remaining samples were divided into training (*n* = 125) and test (*n* = 52) sets at a 70:30 ratio for further analysis. In the training set, significantly differentially expressed miRNAs (adjusted *p* < 0.01) were identified after correcting for multiple testing using a false discovery rate. Subsequently, a predictive classification model using an eight-miRNA signature and a Bayesian logistic regression algorithm was developed. Based on the receiver operating characteristic (ROC) curve analysis in the test set, the model could achieve an area under the curve (AUC) of 0.9542. Together, this study demonstrates the potential use of circulating miRNAs as an adjunct test to stratify breast lesions in patients with abnormal screening mammograms.

## 1. Introduction

Mammography is the current gold standard for breast cancer screening; however, this modality produces false-negatives and also has high false-positive rates [1,2,3,4,5]. According to a study in the United States (U.S.), out of all screening mammograms (*n* = 702,154), approximately 24.5% (*n* = 171,829) were found to be abnormal, with 2,599 women diagnosed with ductal carcinoma in situ (DCIS) or invasive breast cancer [1]. Hence, only 1.5% (2,599 out of 171,829) of women with abnormal mammograms had a breast cancer diagnosis, while the remaining women were unnecessarily recalled for additional diagnostic imaging tests and/or tissue biopsies, which are invasive, expensive, time-consuming, and stress-inducing. Furthermore, as an individual woman may undergo several breast screenings in her lifetime, the cumulative probability of false-positive recalls after 10 years of annual mammogram screening is estimated to be 61% [6]. False-positive mammograms also impact the economy by imposing an exorbitant economic burden. For instance, the national expenditure of false-positive mammograms in the U.S. (including various service costs and fees for follow-up diagnostic procedures) is estimated to be US$2.8 billion annually based on the assumption of an 11% false-positive rate among 29.5 million women aged 40 to 59 years [1].

There is an unmet clinical need for a non-invasive, quick, affordable, and reliable test to differentiate between malignant and benign breast lesions in order to avoid subjecting patients with abnormal mammograms to unnecessary follow-up diagnostic procedures. Liquid biopsies utilizing biomarkers from body fluids [7] are deemed as a promising detection method. However, currently, there are no blood-based biomarkers for the early detection of breast cancer or the stratification or differential diagnosis of breast lesions. Cancer antigen 15-3 (CA15-3), cancer antigen 27-29 (CA27-29), and carcinoembryonic antigen (CEA), which are the only blood tumor markers for breast cancer, are not recommended by the European Group on Tumor Markers (EGTM) and the American Society of Clinical Oncology (ASCO) guidelines for such purposes due to their lack of sensitivity and the insufficient evidence of their roles in early-stage breast cancer management [8,9].

In the last decade, various efforts have been made to discover circulating biomarkers for breast cancer, including microRNAs (miRNAs) [10]. MiRNAs are evolutionary conserved, single-stranded non-coding RNAs (with 19 to 25 nucleotides) with a primary function in mediating the degradation or translational repression of mRNA targets [11]. Apart from its critical roles in the normal physiological state, dysregulated miRNAs are also associated with the hallmarks of cancer through their tumor suppressor or oncogenic activities, and the aberrant expression of miRNAs has been demonstrated in a variety of malignancies [12,13,14]. Circulating miRNAs have been proposed as clinically useful biomarkers not only due to their expression levels changes that are associated with cancer development, but also attributable to their stability in different body fluid sources and extreme conditions (e.g., RNAse digestion, multiple freeze–thaw cycles, and prolonged storage period) [11,13].

Hence, in this study, we aim to identify a miRNA signature with high discriminative ability between malignant and benign breast lesions. Our approach utilized next-generation sequencing (NGS) of 2,083 miRNA transcripts, followed by the identification of significantly differentially expressed miRNAs and the development of a miRNA-based classification model for malignant breast lesions.

## 2. Results

### 2.1. Study Subject Characteristics

The training set had a total of 125 samples comprising of 80 malignant cases and 45 benign controls. The test set had 52 samples with 34 malignant cases and 18 benign controls. Both the training and test sets comprised of various races including Chinese, Malay, Indian, and others. The median ages of the samples in the training and test sets were 55.0 and 53.5 years, respectively. For the malignant cases, both the training and test sets had similar histological types, receptor status, tumor size, tumor grade, and lymph node status (Table 1). Likewise, the training and test sets for the benign breast lesions had similar characteristics (Table 1). Benign lesions with more than one histological pathology (e.g., atypical ductal hyperplasia (ADH) and fibroadenoma (FA)) were counted more than once (Table 1).

### 2.2. Identification of Significantly Differentially Expressed Serum Circulating MiRNAs from NGS Profiling

Comprehensive profiling of 2,083 miRNAs was carried out employing the HTG EdgeSeq miRNA whole transcriptome assay on a cohort of 180 subjects comprising 116 malignant cases and 64 benign controls. Three outliers (2 malignant cases and 1 benign control) were excluded from the dataset based on the principal component analysis (PCA; Figure S1) in order to limit skewing of the dataset, which would otherwise lead to biasness in data analysis or misleading analysis outcomes. The remaining samples were randomly divided into training (*n* = 125) and test (*n* = 52) sets.

**Figure 1:**
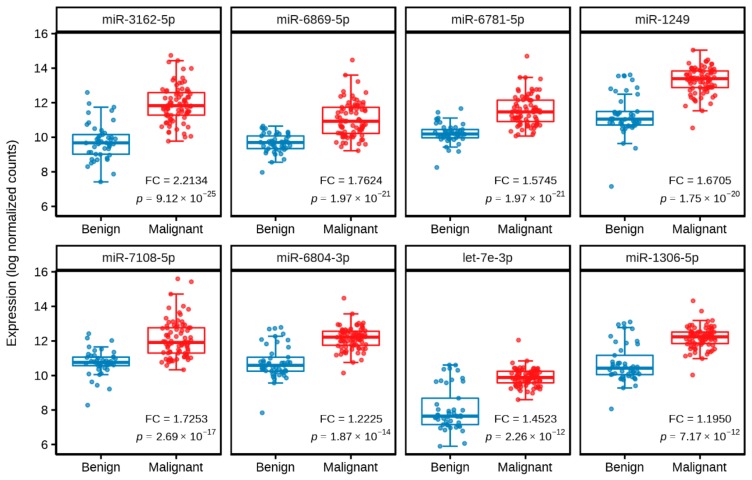
Boxplot of differentially expressed miRNAs for malignant cases versus benign controls in the training set. The expression levels of miRNAs in malignant and benign breast lesions are represented in log normalized counts, with horizontal lines indicating mean and standard deviation. FC denotes fold change (log_2_), and *p* denotes adjusted *p*-value.

Using the training set, eight training subsets were generated and differential expression analysis was performed on each of the training subsets. Following the analysis, different numbers of significantly differentially expressed miRNAs (which ranged from 19 to 44 miRNAs) with an absolute log_2_ fold change of ≥1 and a false discovery rate (FDR) < 0.01 were obtained from the eight training subsets (data not shown). Of these significantly differentially expressed miRNAs, eight miRNAs (miR-3162-5p, miR-6869-5p, miR-6781-5p, miR-1249, miR-7108-5p, miR-6804-3p, let-7e-3p, and miR-1306-5p) were consistently found in all eight training subsets. In the training set, these eight miRNAs were significantly upregulated in malignant cases as compared to benign controls (*p* < 1 × 10^−11^; Table 2; Figure 1).

**Figure 2:**
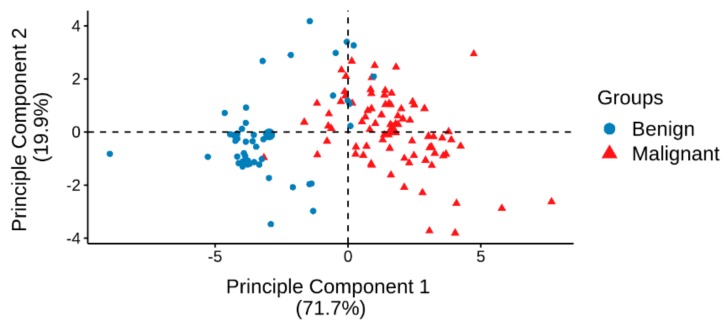
Principal component analysis (PCA) plot of malignant cases and benign controls from the training set generated using eight significantly differentially expressed miRNAs.

Next, PCA with the eight significantly differentially expressed miRNAs was used to visualize their discrimination levels between malignant cases and benign controls. A clear separation was observed between malignant cases and benign controls (Figure 2).

### 2.3. Development of Classification Model and Receiver Operating Characteristic (ROC) Curve Analysis

**Figure 3:**
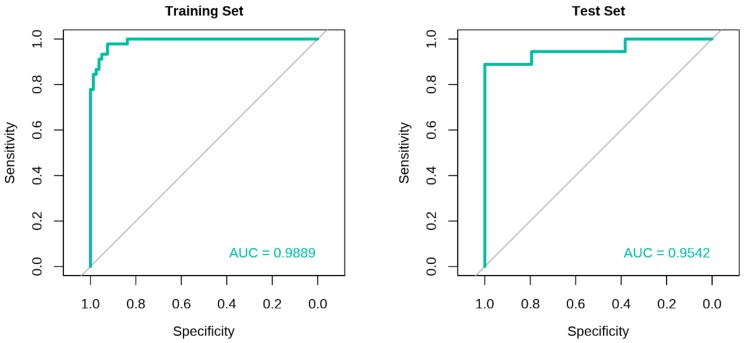
Receiver operating characteristic (ROC) curves for the eight-miRNA signature. The ROC curves of the training and test sets showing the AUCs obtained using a Bayesian logistic regression model.

A Bayesian logistic regression model was created using the eight miRNAs discovered from the training set. The training model yielded an area under the curve (AUC) of 0.9889 based on the ROC curve analysis, with recall, precision, and balanced accuracy of 0.9625, 0.9506, and 0.9368, respectively (Figure 3, Table 3). The model was tested using an independent test set, which resulted in an AUC, recall, precision, and balanced accuracy of 0.9542, 0.9412, 0.9412, and 0.9150, respectively (Figure 3, Table 3). Of the 52 subjects from the test set, 48 were correctly classified, and four were misclassified. Among the misclassified subjects, two were false-negatives (both were DCIS), and two were false-positives (one had FA, whereas another had fibrocystic change with residual lobular carcinoma in situ (LCIS)).

## 3. Discussion

This present study describes the identification of a circulating eight-miRNA signature which was significantly differentially expressed between malignant breast lesions (cases) and benign breast lesions (controls), and could segregate both cases and controls on PCA. In addition, the model showed high precision, recall, and balanced accuracy values. Together, the present findings demonstrate the use of circulating miRNAs from blood as a promising tool to detect breast cancer with higher AUCs in discriminating malignant cases and benign controls as compared to previous miRNA discovery studies [15,16,17].

Our miRNA biomarker signature, which had an AUC, recall, precision, and balanced accuracy of 0.9542, 0.9412, 0.9412, and 0.9150, respectively, has the potential to be further developed into a blood test for the stratification of breast lesions. Such a blood test could be incorporated into the diagnostic/clinical workflow (i.e., after the initial screening mammography but before the diagnostic imaging assessments and tissue biopsy) with the aim of assisting clinicians in their decision making. For example, a negative test result could potentially indicate that the initial screening mammogram is a false-positive; hence, no further diagnostic imaging procedures or tissue biopsy would be warranted. In contrast, a positive test result would lead to additional diagnostic imaging and/or a tissue biopsy being performed in order to confirm the presence or absence of malignancy in the breast lesion.

A wide range of blood-based biomarker candidates, including DNA, RNA, protein, methylation, lipid, and metabolites, have been reported in the literature, with some showing notable potential for breast cancer detection [10]. Some key studies are the CancerSEEK [18] and GRAIL’s Circulating Cell-Free Genome Atlas (CCGA) [19,20] projects that have employed circulating DNAs as cancer markers. Although these circulating DNA-based blood tests were able to identify multiple cancers, their sensitivities in discriminating breast cancer from healthy or non-cancer controls remained poor (i.e., 33% for CancerSEEK [18] and <60% for CCGA [20]). Besides, these tests were less sensitive in detecting breast cancer at early stages as compared to late stages [19,20]. Other potential biomarkers for differentiating breast cancers and benign breast lesions include protein biomarkers such as the combination of epithelial membrane antigen and cytokeratin-1 (EMA and CK1; AUC of 0.9010), and developmental endothelial locus-1 (Del-1; AUC of 0.9200) [21,22]. The discriminative ability of individual methylated promoters of adenomatous polyposis coli (*APC*) and retinoic acid receptor β_2_ (*RARβ_2_*) in classifying malignant and non-malignant controls yielded AUCs above 0.9400, and this outperformed traditional tumor markers (CEA and CA15-3) in identifying low-grade tumors, early-stage and triple-negative breast cancers [23].

In addition, there are other significant miRNA biomarker candidates that have been published. The study by Shimomura et al. (2016) is the largest breast cancer miRNA discovery to date, which reported a five-miRNA signature—miR-1246, miR-1307-3p, miR-4634, miR-6861-5p, and miR-6875-5p—that could recognize breast cancer cases from non-cancer controls at an AUC of 0.9710 [24]. Nevertheless, the downside of the study is that the biomarker signature was profiled and developed using a microarray-based platform which is known to have poor specificity [25], and only one of the five miRNAs could be validated in 26 serum samples by qRT-PCR.

In another study, an efficient artificial neural network (ANN) has been developed to identify malignant breast lesions in women with the Breast Imaging and Reporting Data System (BI-RADS) 4 mammography following screening (*n* = 118), validation (*n* = 58), and testing phases (*n* = 62) [26]. It was shown that the ANN could discriminate the BI-RADS 4 breast lesions as malignant or benign using the plasma levels of miR-15a, miR-101, and miR-144 at an AUC of 0.9603 [26]. In a more recent study using qRT-PCR, serum miR-126, miR-155, and miR-21 have been identified as individual markers for distinguishing breast cancer cases (*n* = 96) from patients with benign breast lesions (*n* = 47) or healthy individuals (*n* = 39), with AUCs of 0.9980, 0.9950, and 0.8570, respectively [27]. However, these findings have yet to be validated. Besides miRNAs, new signatures derived from metabolites and lipids capable of discriminating between malignant and benign breast lesions have emerged [28,29]. Of note, the present study’s miRNA signature shows comparable AUCs to the abovementioned studies.

The eight miRNAs identified in our present signature comprised of let-7e-3p, miR-1249, miR-6869-5p, miR-3162-5p, miR-1306-5p, miR-6781-5p, miR-6804-3p, and miR-7108-5p which were upregulated in malignant breast lesions as compared to benign breast lesions. The roles of these miRNAs have not been widely characterized and require further elucidation due to limited publications in the literature. Of the eight miRNAs, only let-7e-3p and miR-1249-3p have been associated with breast cancer. Let-7e-3p has been identified as one of the miRNAs involved in breast cancer development that is regulated by C-terminal binding protein 1 (CTBP1) and metabolic syndrome [30]. Besides, let-7e-3p has been suggested to play a tumor suppressor role especially in estrogen receptor (ER)-negative or basal-like tumors [31], and low expression of let-7e-3p in breast cancer tissues has been associated with poor prognosis [31]. However, in the present study, the upregulation of let-7e-3p expression was observed in cases with malignant breast lesions. For miR-1249-3p, it has been shown that the overexpression of miR-1249-3p in breast cancer cell lines suppressed cell migration, proliferation, and epithelial-mesenchymal transition [32]. Furthermore, the miR-1249-3p/Homeobox B8 (HOXB8) axis was involved in long non-coding RNA MIF-AS1-mediated breast cancer progression [32].

Apart from breast cancer, miR-1249-3p alongside with miR-6869-5p and miR-3162-5p have been implicated in other malignancies. The upregulation of miR-1249-3p promoted glioma cell proliferation by downregulating the expression of a key regulator of the Wnt/β-catenin signaling pathway, the adenomatous polyposis coli 2 (APC2) [33]. In colorectal cancer, miR-1249-3p may present as a novel therapeutic candidate based on its inhibitory actions in tumor growth, metastasis, and angiogenesis by targeting vascular endothelial growth factor A (VEGFA) and high mobility group AT-hook 2 (HMGA2) [34]. Furthermore, miR-1249-3p was one of eight miRNAs that have been proposed to be useful predictors for postoperative outcomes in patients with small cell carcinoma of the esophagus due to its significant correlation with tumor relapse [35]. However, these results should be further validated as only six samples were used in this study—three cases having rapid tumor relapse and three cases with long-term survival [35]. In addition, the involvement of miR-1249-3p has also been described in liver cancer based on two independent studies. The first study reported the downregulated expression of miR-1249-3p in tumor tissues [36], whereas the second study observed that elevated levels of miR-1249-3p in hepatocellular carcinoma tissues from patients were correlated with a poorer prognosis [37]. MiR-6869-5p may act as a tumor suppressor and has been identified as a potential prognostic biomarker for colorectal cancer [38]. Decreased levels of miR-6869-5p in serum exosomes were reported to be associated with a poor 3-year survival period among colorectal cancer patients [38].

In addition, the associations of miR-3162-5p to cervical and prostate cancers have been reported. For patients with early-stage cervical squamous cell carcinoma, miR-3162-5p was a member of a six-miRNA panel that was able to predict lymph node metastasis with an AUC of 0.9320 [39]. In prostate cancer, a probable role of miR-3162-5p in the mechanism underlining the increased prostate cancer risk with the kallikrein-related peptidase 3 (KLK3) rs1058205 miRSNP T-allele has been demonstrated [40]. Furthermore, in a recent study, the increased expression of miR-3162-5p in prostate tumor tissues has been associated with a higher Gleason grade; thus, indicating the possible use of miR-3162-5p in stratifying patients who are at risk of developing aggressive prostate cancer [41].

Although miR-1306-5p and miR-6781-5p have not been previously reported to be involved in any cancers, aberrant expression of these miRNAs in plasma samples have been detected in patients with conditions such as heart failure [42,43], glaucoma [44], or epilepsy [45]. On the other hand, miR-6804-3p was identified as one of the miRNA candidates for antiviral therapeutics against the Middle East respiratory syndrome (MERS) based on a computational approach using bioinformatics tools [46], whereas no information on miR-7108-5p and its disease association has been reported in the literature.

Despite the plethora of potential breast cancer biomarkers described in the literature, none have proceeded to clinical trials [10]. To enhance the translatability of our current findings into actual clinical practice, several gaps and limitations of prior studies have been taken into consideration and addressed in the present study. To the best of our knowledge, this is the most comprehensive miRNA profiling for breast cancer that has been conducted thus far, with a total of 180 malignant and benign breast lesions screened for the expression of 2,083 miRNA transcripts. To date, only a few cancer studies have deployed NGS as a tool in circulating miRNA biomarker discovery [47,48], with none reported for breast cancer. NGS was chosen as the profiling platform in this study due to its high-throughput capabilities and superior detection sensitivity as compared to other methods such as microarray and real-time qRT-PCR. Besides that, the present work is the only biomarker discovery study for breast cancer that has utilized multi-racial and multi-center Asian cohorts with pre-operative serum blood samples prospectively collected from women with abnormal mammograms. To ensure a minimal effect of confounding factors and technical biasness in data analysis, pre-analytical factors were standardized in terms of sample collection, handling, processing, and storage [49]. Furthermore, the training and test sets encompassed independent study cohorts to prevent overfitting in machine learning.

Nonetheless, there are limitations in the present study. Firstly, although the present miRNA signature showed promise in discriminating malignant and benign breast lesions, there is still a need to validate these results in independent cohorts of cases and controls. It has been well-documented that previous breast cancer circulating miRNA biomarker studies published by independent investigators showed inconsistent results and a lack of overlapping findings [50,51]. These inconsistencies could be attributed to technical biasness or constraints in the study design, or failure to properly assess and account for individual or environmental factors during data analysis [49,52]. In addition, validation studies such as prospective cohort studies or blinded studies, are also warranted to examine the clinical validity of the present miRNA signature and to benchmark against the existing imaging modalities [10]. With prospective cohort studies, the clinical utility of the miRNA signature can also be determined by comparing the diagnostic outcome as a result of using the miRNA signature to the outcome as a result of not using it [53]. In addition, the sensitivity and the specificity of the miRNA signature should also be tested in blood samples obtained from other ethnic populations such as Caucasians, as well as blood samples from subjects of other cancer types. Secondly, our model misclassified four out of 18 benign breast lesions. Two DCIS cases were misclassified as having benign lesions, whereas two benign controls (one with FA, and another with fibrocystic change and LCIS) were misclassified as having malignant lesions. These misclassifications could be due to underlying biological similarities in malignant and benign lesions [54]. The two subjects with benign lesions could be monitored closely to determine if they develop breast cancers in the future [55,56]. Thirdly, it is plausible that comorbidities and medications taken by subjects could affect the expression levels of circulating miRNAs. Numerous circulating miRNA biomarker studies lack adjustment for these factors. Hence, for future research, adequate consideration should be given to account and adjust for the possible effects of comorbidities and medications in the expression of circulating miRNA biomarkers.

Together, the present study highlights the discovery of an eight-miRNA signature as a novel circulating biomarker for breast cancer. Based on this miRNA signature, we have generated a classification model with high discrimination ability in classifying malignant and benign breast lesions at an AUC of 0.9542. The present miRNA signature may have the potential to be developed as an adjunct test to stratify breast lesions in patients with abnormal screening mammograms, with the aim of reducing the number of patients with false-positive mammograms from being subjected to unwarranted tissue biopsies. For future investigations, additional cohorts for prospective or blinded studies should be carried out to ascertain the clinical validity and clinical utility of this miRNA signature.

## 4. Materials and Methods

### 4.1. Study Subjects

All subjects in this study had initial abnormal screening mammograms, and were evaluated with further diagnostic imaging tests (conducted by specialist radiologists) before being referred for tissue biopsies. The subjects in this study did not include patients with benign lesions or calcifications who were discharged following additional diagnostic imaging tests. Pre-operative peripheral blood samples were prospectively collected from women with abnormal mammograms at the National Cancer Centre Singapore (Singapore, Singapore) and Tan Tock Seng Hospital (Singapore, Singapore) between the years 2015 to 2017. In addition, serum samples were also obtained from the SingHealth Tissue Repository (Singapore, Singapore). There were 82 women with invasive ductal/lobular/mucinous/papillary carcinomas, 34 with DCIS, and 64 with benign breast lesions. In this study, women with invasive carcinomas or DCIS were defined as malignant cases, whereas women with benign breast lesions were defined as benign controls. Written informed consent was obtained from all participants of this study, and ethics approval for this study was obtained from the Centralized Institutional Review Board (CIRB) of SingHealth (CIRB Reference number: 2018/2874).

### 4.2. Serum Samples Processing

All blood samples were collected prior to or during surgery. Blood samples were collected in BD Vacutainer SST tubes (Becton, Dickinson and Company, Franklin Lakes, NJ, USA) and were centrifuged at 1300 rcf for 20 min at room temperature within 50 to 60 min of venipuncture. Serum samples were stored in aliquots at −80 °C until use. Only serum samples without hemolysis were used.

### 4.3. Serum Samples for NGS

Serum samples were thawed on ice, and the samples were prepared according to the HTG EdgeSeq miRNA whole transcriptome assay (HTG Molecular Diagnostics, Inc., Tucson, AZ, USA) protocol by HTG Molecular Diagnostics, Inc. Purified PCR products were obtained according to the manufacturer’s protocol and were subsequently used for sequencing. Human brain reference RNA samples (Ambion, Austin, TX, USA) were processed and served as controls. The libraries were sequenced on the Illumina NextSeq platform (Illumina, San Diego, CA, USA) using a High Output Kit v2 (75 cycles) with two index reads (Illumina). PhiX was spiked into the library at 5%. Data from the sequencer was obtained as demultiplexed FASTQ files. The HTG EdgeSeq Parser was used to align the FASTQ files to miRNA reference sequences obtained from the miRbase20 database. Raw data counts of sequences corresponding to each miRNA were generated and were normalized using the median of ratios method by the DESeq2 tool (version 1.20.0).

### 4.4. Model Development

#### 4.4.1. MiRNA Selection

**Figure 4:**
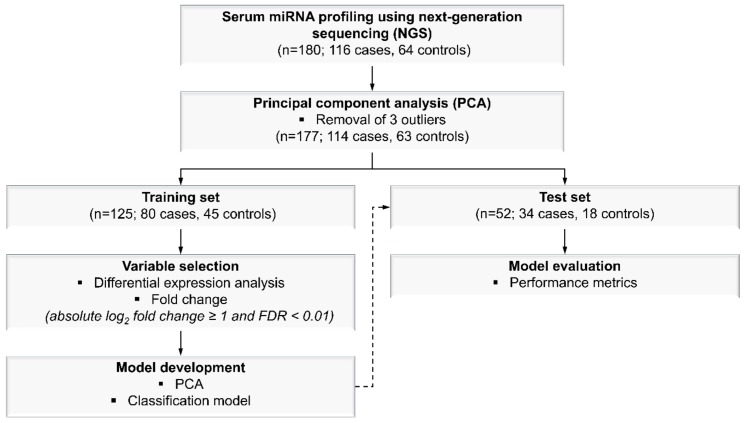
Workflow for miRNA profiling, miRNA selection, and model development for breast cancer. A total of 2,083 miRNA transcripts were profiled from serum samples (*n* = 180) using the next-generation sequencing (NGS)-based method. Following NGS, outliers were removed (*n* = 3) based on principal component analysis (PCA), and the remaining samples were randomly divided into training (*n* = 125) and test (*n* = 52) sets at a 70:30 ratio. The training set was used for variable selection and model development, whereas the test set was subsequently used for model evaluation based on various performance metrics.

PCA was performed on all 180 samples using 2,083 miRNAs to detect outliers from the dataset (Figure 4). After removal of three outliers based on PCA, the samples were randomly and proportionally divided into training (*n* = 125; 80 malignant cases and 45 benign controls) and test (*n* = 52; 34 malignant cases and 18 benign controls) sets in an estimated 70:30 ratio (Figure 4). For miRNA selection, only the training set was used for the purpose of identifying miRNAs that could discriminate between malignant cases and benign controls. From the training set, eight training subsets were generated; each training subset comprised of different sample sizes ranging from 30% to 100% of the total number of samples in the training set, respectively. The selection of samples in the training subsets were random. In each training subset, differential expression analysis was performed using the DESeq2 tool (version 1.20.0) [57]. Significantly differentially expressed miRNAs were defined as miRNAs with an absolute log_2_ fold change of ≥1 and an FDR < 0.01 for malignant cases versus benign controls. A reason for performing this analysis on the eight training subsets separately was to identify significantly differentially expressed miRNAs that were common in all eight training subsets in order to eliminate the influence of sample size in the selection of miRNAs for further analysis.

#### 4.4.2. Classification Model Generation for Malignant and Benign Breast Lesions

To develop a classification model for malignant and benign breast lesions, the miRNA signature, which comprised of the significantly differentially expressed miRNAs identified in the training set, was used as input variables to the statistical algorithms. An unsupervised clustering based on PCA was performed using the miRNA signature, and the first two principal components were used to visualize the clustering of the malignant cases and benign controls. In order to develop a supervised-learning model using the training set, the repeated k-fold cross validation technique (k = 10, repeated five times) and the Bayesian logistic regression algorithm were applied. The performance of the classification model that was built using the training set was evaluated using an independent test set based on different evaluation metrics including specificity, sensitivity, and AUC of the ROC curve. In addition, recall and precision metrics were also estimated due to the imbalanced proportion between malignant cases and benign controls. All statistical and machine-learning analysis were carried out using R software (version 3.5.1). The R caret package (version: 6.0.84) was used to train, develop, and test the performance of the model, whereas the ROC curves were generated using the R pROC package (version 1.15.3).

## 5. Conclusions

The present study reports the discovery of a serum-derived eight-miRNA signature, comprising of miR-3162-5p, miR-6869-5p, miR-6781-5p, miR-1249, miR-7108-5p, miR-6804-3p, let-7e-3p, and miR-1306-5p. This novel circulating biomarker signature showed promise in discriminating between malignant and benign breast lesions among women with abnormal screening mammograms. These current findings suggest a possible use of circulating miRNAs as an adjunct test for the stratification of breast lesions, with the potential to reduce the number of unwarranted tissue biopsies among those with abnormal screening mammograms.

## Figures and Tables

**Table 1:** Clinico-pathological characteristics of malignant cases and benign controls.

Clinico-Pathological Characteristics	Training Set (*n* = 125)	Test Set (*n* = 52)
**Age ^a^**		
Mean	56.0	55.2
Median	55.0	53.5
Range	34–87	39–79
**Race**		
Chinese	108 (86.4%)	42 (80.8%)
Malay	8 (6.4%)	1 (1.9%)
Indian	7 (5.6%)	6 (11.5%)
Others	2 (1.6%)	3 (5.8%)
**Malignant Cases**	80	34
**Age at diagnosis**		
Mean	57.6	58.2
Median	57.5	58.0
Range	34–87	41–79
**Histological type**		
Invasive ductal carcinoma (IDC)	37 (46.3%)	18 (52.9%)
Invasive lobular carcinoma (ILC)	3 (3.8%)	1 (2.9%)
Ductal carcinoma in-situ (DCIS)	26 (32.5%)	8 (23.5%)
Others ^b^	6 (7.5%)	3 (8.8%)
Mixed		
IDC with another histological type	7 (8.8%)	4 (11.8%)
Others ^c^	1 (1.3%)	nil
**Receptor status**		
Estrogen receptor (ER)		
Positive	70 (87.5%)	28 (82.4%)
Negative	10 (12.5%)	5 (14.7%)
Unknown	nil	1 (2.9%)
Progesterone receptor (PR)		
Positive	54 (67.5%)	23 (67.6%)
Negative	25 (31.3%)	10 (29.4%)
Unknown	1 (1.3%)	1 (2.9%)
Human epidermal growth factor receptor 2 (HER2)		
Positive	13 (16.3%)	4 (11.8%)
Negative	33 (41.3%)	12 (35.3%)
Equivocal	10 (12.5%)	10 (29.4%)
Not tested (DCIS) ^d^	20 (25.0%)	8 (23.5%)
Unknown	4 (5.0%)	nil
**Tumor size**		
<20 mm	44 (55.0%)	17 (50.0%)
20 mm to 50 mm	30 (37.5%)	15 (44.1%)
>50 mm	4 (5.0%)	2 (5.9%)
Unknown	2 (2.5%)	nil
**Tumor grade**		
Invasive carcinomas		
Grade 1	7 (8.8%)	7 (20.6%)
Grade 2	27 (33.8%)	9 (26.5%)
Grade 3	18 (22.5%)	10 (29.4%)
Unknown	2 (2.5%)	nil
DCIS		
High nuclear grade	10 (12.5%)	1 (2.9%)
Intermediate nuclear grade	12 (15.0%)	4 (11.8%)
Low nuclear grade	4 (5.0%)	3 (8.8%)
**Lymph node status**		
Positive	20 (25.0%)	9 (26.5%)
Negative	44 (55.0%)	21 (61.8%)
Not tested (DCIS) ^d^	11 (13.8%)	3 (8.8%)
Unknown	5 (6.3%)	1 (2.9%)
**Benign Controls**	45	18
**Age at diagnosis**		
Mean	53.1	49.5
Median	52.0	47.0
Range	40–82	39–66
**Type of lesion ^e^**		
Atypical ductal hyperplasia (ADH)	2 (4.4%)	nil
Lobular carcinoma in-situ (LCIS)	1 (2.2%)	1 (5.6%)
Fibrocystic changes	17 (37.8%)	6 (33.3%)
Sclerosing adenosis	2 (4.4%)	2 (11.1%)
Moderate or florid ductal hyperplasia of the usual type	2 (4.4%)	nil
Radial scar	1 (2.2%)	nil
Fibroadenoma (FA)	21 (46.7%)	11 (61.1%)
Others	10 (22.2%)	3 (16.7%)

^a^ Age refers to the age of breast cancer diagnosis for cases and the age at the point of study recruitment for controls. ^b^ Other histological types include cases with invasive mammary carcinoma, invasive mucinous carcinoma, invasive papillary carcinoma, and metaplastic squamous cell carcinoma. ^c^ This single case was diagnosed with both invasive micropapillary carcinoma and malignant phyllodes. ^d^ The majority of DCIS cases did not undergo HER2 and lymph node testing. ^e^ The samples with benign lesions are more often than not diagnosed with multiple histological types (e.g., both ADH and FA), thus, many of the benign lesions have been counted more than once in this list.

**Table 2:** Differentially expressed miRNAs in malignant cases versus benign controls in the training set.

MiRNA	Fold Change (log_2_)	Adjusted *p*-Value	Expression
miR-3162-5p	2.2134	9.12 × 10^−25^	Upregulation
miR-6869-5p	1.7624	1.97 × 10^−21^	Upregulation
miR-6781-5p	1.5745	1.97 × 10^−21^	Upregulation
miR-1249	1.6705	1.75 × 10^−20^	Upregulation
miR-7108-5p	1.7253	2.69 × 10^−17^	Upregulation
miR-6804-3p	1.2225	1.87 × 10^−14^	Upregulation
let-7e-3p	1.4523	2.26 × 10^−12^	Upregulation
miR-1306-5p	1.1950	7.17 × 10^−12^	Upregulation

**Table 3:** Performance of the miRNA-based classification model to distinguish malignant and benign breast lesions.

Performance Metrics	Eight-MiRNA Signature Model	
	Training Set	Test Set
AUC (95% CI)	0.9889 (0.9772, 1.0000)	0.9542 (0.8832, 1.0000)
Recall	0.9625	0.9412
Precision	0.9506	0.9412
Balanced Accuracy	0.9368	0.9150

## Supplementary Materials

The following are available online at https://www.mdpi.com/2072-6694/11/12/1872/s1, Figure S1: Principal component analysis (PCA) plot of all 180 samples using 2083 miRNAs.
