# Supplementary material for: A Circulating miRNA Signature for Stratification of Breast Lesions among Women with Abnormal Screening Mammograms
Source: Cancers (Basel). 2019 Nov 26;11(12):1872. doi: 10.3390/cancers11121872 (PMC6966622; doi:10.3390/cancers11121872)
Supplement: Supplementary file 1 [file cancers-11-01872-s001.pdf]

cancers

Supplementary Materials

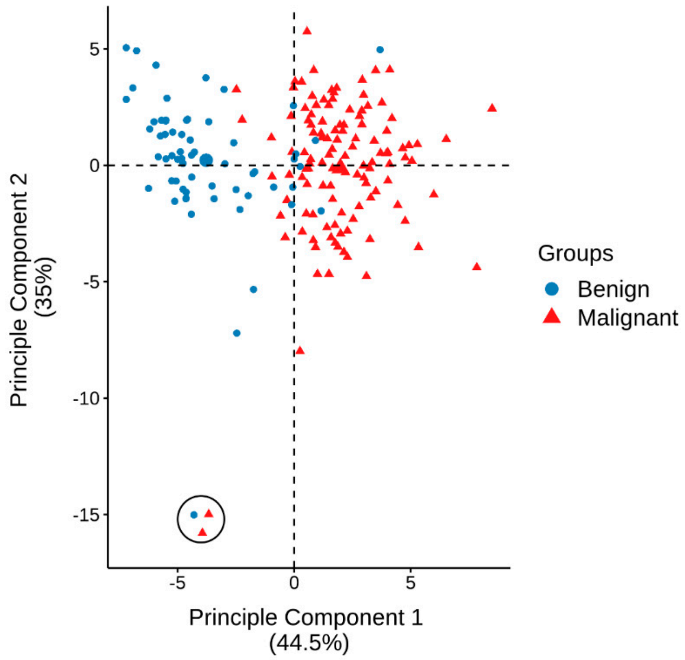

Figure S1. Principal component analysis (PCA) plot of all 180 samples using 2,083 miRNAs. The samples within the circle indicate three outliers (2 malignant cases and 1 benign control) observed in the dataset.
